# Rosmarinic acid induces programmed cell death in Arabidopsis seedlings through reactive oxygen species and mitochondrial dysfunction

**DOI:** 10.1371/journal.pone.0208802

**Published:** 2018-12-26

**Authors:** Fabrizio Araniti, Aitana Costas-Gil, Luz Cabeiras-Freijanes, Antonio Lupini, Francesco Sunseri, Manuel J. Reigosa, Maria Rosa Abenavoli, Adela M. Sánchez-Moreiras

**Affiliations:** 1 Department AGRARIA, University Mediterranea of Reggio Calabria, Feo di Vito, Reggio Calabria, Italy; 2 Department of Plant Biology and Soil Science. University of Vigo. Campus Lagoas-Marcosende, Vigo, Spain; 3 CÍTACA. Agri-Food Research and Transfer Cluster, Campus da Auga. University of Vigo, Ourense, Spain; University of South Alabama Mitchell Cancer Institute, UNITED STATES

## Abstract

Phytotoxic potential of rosmarinic acid (RA), a caffeic acid ester largely found in aromatic species, was evaluated on Arabidopsis through metabolomic and microscopic approaches. *In-vitro* bioassays pointed out that RA affected root growth and morphology, causing ROS burst, ROS scavengers activity inhibition and consequently, an alteration on cells organization and ultrastructure. In particular, RA-treatment (175 μM) caused strong vacuolization, alteration of mitochondria structure and function and a consistent ROS-induced reduction of their transmembrane potential (ΔΨ_m_). These data suggested a cell energy deficit also confirmed by the metabolomic analysis, which highlighted a strong alteration of both TCA cycle and amino acids metabolism. Moreover, the increase in H_2_O_2_ and O_2_^−^ contents suggested that RA-treated meristems underwent oxidative stress, resulting in apoptotic bodies and necrotic cells. Taken together, these results suggest that RA inhibits two of the main ROS scavengers causing high ROS accumulation, responsible of the alterations on mitochondrial ultrastructure and activity through ΔΨ_m_ dissipation, TCA-cycle alteration, cell starvation and consequently cell death on Arabidopsis seedlings. All these effects resulted in a strong inhibition on root growth and development, which convert RA in a promising molecule to be explored for further use in weed management.

## Introduction

The use of natural compounds, generally belonging to the secondary plant metabolism, as bioherbicides or backbone for novel agrochemicals, is becoming a suitable alternative to the synthetic herbicides for an environmental friendly control of weeds [1,2]. Indeed, natural compounds usually affect weed growth by acting at different physiological and biochemical levels [3] and, although their efficacy and specificity are limited, they generally do not have residual or toxic effects [4]. Furthermore, their mutiple targets ability allows us to overcome the limit of most of herbicides that, interfering with a single molecular site, inhibit specific biochemical processes causing a rapid evolution of resistance to these molecules [5,6]. Therefore, novel agrochemicals with new modes of action (MOAs) and multiple target activities are strongly required to counter the increased herbicide resistance [7].

Many allelochemicals influence cell ultra-structure, cell division and elongation, membrane permeability, growth regulation systems, respiration, enzyme synthesis and metabolism, photosynthesis, protein and nucleic acid synthesis [8–11]. They are also known to be stress inducers in acceptor plants causing metabolic changes, oxidative stress and alteration in mineral ion uptake [8,12,13]. This wide biological activity explains the important role that secondary metabolites can play in the future agriculture. During the last years, different natural compounds have been developed and used as bioherbicides as an alternative strategy to ‘conventional’ synthetic herbicides. Most of the active substances of these bioherbicides are generally secondary metabolites similar to rosmarinic acid, i.e. pelargonic acid, carvacrol, eugenol, etc, which are being successfully used in weed control [14].

For example, hydroxycinnamic acid and their derivatives have been largely studied for their phytotoxic potential and role in plant-plant interaction [15,16], acting as potential growth regulators, insecticides, and antimicrobial crop protection products [2].

Rosmarinic acid (RA), an ester of caffeic acid and 3,4-dihydroxyphenyllactic acid, is a natural compound occurring in species of the Boraginaceae and Lamiaceae (subfamily Nepetoideae) families, as well as in other higher plant families and in some fern species [17].

This compound has been largely studied for its wide biological activity, which includes antiviral, antibacterial, anti-mutagen, anti-inflammatory and antioxidant properties [17]. It seems to act as a constitutively accumulated defense compound [18], although its toxicity was demonstrated on the diatom *Phaeodactylum tricornutum*, whose survival was affected by a RA-induced alteration of plasma membrane permeability [19].

Despite the large number of evidences regarding to RA pharmacological activity and its role on plant-microorganism interaction [20], scarce information is available on both its phytotoxic potential and its mode of action on plants [21].

Therefore, the aim of this work was to identify the potential mode of action of rosmarinic acid on *Arabidopsis thaliana* through a multidisciplinary approach. In particular, a physiological, cytological and metabolomic approach was used to identify the effects of this secondary metabolite and to elucidate its mechanism of action on plant metabolism.

## Materials and methods

### Bioassays on *Arabidopsis thaliana*

Rosmarinic acid (RA) (Sigma Aldrich, Italy) was firstly dissolved in 0.1% EtOH (v:v) and then diluted in deionized water to reach the final concentrations: 0, 50, 100, 200, 400, 800 and 1200 μM.

To evaluate the phytotoxic effect of RA on root morphology, seeds of *Arabidopsis thaliana* (L.) Heynh, ecotype Columbia (Col-0), were sterilized and then germinated on Petri dishes (100 x 150 mm) containing agar medium (0.8% w/v), enriched with micro- and macronutrients (Murashige-Skoog, Sigma-Aldrich) and supplemented with 1% sucrose, as previously described by Araniti et al. [22]. The Petri plates were then transferred to a growth chamber in a vertical position at 22 ± 2°C temperature, 75 μmol m^-2^ s^-1^ light intensity, 55% relative humidity, and 8/16 h light/dark. Immediately after germination, five seedlings, per replicate and treatment, were transferred to Petri dishes containing the same medium with RA added at concentrations reported above. After 14 days of treatment, whole root system was imaged by scanning (STD 1600, Régent Instruments Inc., Quebec, Canada) and Total Root Length (TRL), Primary Root Length (PRL), Number of Lateral Roots (NLR) and Lateral Root Length (LRL) were measured using WinRhizo Pro system v. 2002a (Instruments Régent Inc., Quebec, Canada). Root Hair Density (RHD) and Root Hair Length (RHL) were analysed by using a stereoscopic microscope (Olympus SZX9) and the software Image Pro Plus v 6.0 (Media Cybernetics).

An additional experiment was carried out on seedlings treated with sodium azide (NaN_3_) (used as positive control), a known superoxide dismutase (SOD) and catalase (CAT) inhibitor (ED_50_ = 1 mM and 25 μM, respectively) [23,24,25,26,27], using the following concentrations: 0, 25, 50, 100, 200, 400 and 800 μM.

### In situ semi-quantitative detection of H_2_O_2_ and O_2_^−^

Root tips of seedlings untreated (control) and treated with 175 μM RA, for 7 and 14 days, were cut, transferred to distilled water and vacuum infiltrated for 5 min with 0.65 mg mL^−1^ NaN_3_ solution in potassium phosphate buffer (pH 7.8) containing 0.1% (w/v) nitroblue tetrazolium (NBT), for superoxide (O_2_^-^) detection. For *in situ* hydrogen peroxide (H_2_O_2_) localization, root tips were transferred to acidified water (pH 3.8) containing 3,3-diaminobenzidine (DAB) [13]. After infiltration, roots were incubated in darkness for 20 min and, successively, illuminated until the typical appearance of reddish-brown and dark blue color spots, for DAB (H_2_O_2_) or NBT (O_2_^-^) staining, respectively.

### Catalase (CAT) and superoxide dismutase (SOD) activities and lipid peroxidation

Catalase and SOD activities were evaluated using 500 mg of fresh root material from seedlings untreated (control) and treated with 175 μM RA, for 7 and 14 days. Roots were extracted using an extraction buffer in a 1:4 ratio (1 part of root material and 4 parts of buffer) at pH 7, composed by NaH_2_PO_4_ (50 μM), 2-mercaptoethanol (5 mM), dithiothreitol (2 mM), ethylenediaminetetraacetic acid disodium salt (2 mM) and polyvinylpyrrolidone (1% w/v). All the extraction steps were carried out at 4°C. The sample was then centrifuged at 14.000 rpm (4°C) for 10 min, and the supernatant was collected for the evaluation of SOD and CAT activities according to Elstener et al. [28] and Aebi [29], respectively. Protein content was measured by the Bradford method [30].

To evaluate a direct effect of RA on SOD and CAT, an *in-vitro* experiment was carried out extracting both enzymes from 14 days old untreated plants and adding RA (0 μM and 175 μM) before spectrophotometer measurement. Enzymes extraction and activity, and protein content were carried out as previously described.

Lipid peroxidation, indirectly determined by measuring the content of malondialdehyde (MDA), was evaluated according to the protocol of Hodges et al. [31], with some modifications. Root material (50 mg), previously powdered in liquid nitrogen, was homogenized with 1 mL of 80% ethanol and then centrifuged at 3000 rpm at 4°C, for 10 min. The supernatants were then incubated for 25 min at 95°C with 20% TCA containing 0.01% hydroxytoluenebutylate, with and without 0.5% thiobarbituric acid (TBA). The reaction was then stopped in ice. Samples were again centrifuged at 3000 rpm at 15°C, for 10 min, and the absorbance measured at 440, 532 and 600 nm. MDA equivalents were calculated using the equations proposed by Hodges et al. [31]. All data were finally expressed as percentage of the control.

### Transmission electron microscopy (TEM) for ultrastructural root studies

Ultrastructural studies of Arabidopsis root tips untreated and treated with 175 μM RA, for 7 and 14 days, were carried out as previously described by Graña et al. [11]. Forty root tips per replicate were cut and immersed in 0.1 M cacodylate buffer (pH 7.2), containing glutaraldehyde fixative (5%), for 4 h. After incubation, root tips were washed 3 times (4 h each) with 0.1 M cacodylate buffer (pH 7.2). Successively, samples were immersed in 0.1 M cacodylate buffer enriched with osmium tetroxide (2%) for 3 h, and then in 10% acetone with 2% uranyl acetate, for 1 h. The dehydration of the sample was carried out through consecutive immersions in acetone at different concentrations: 50% acetone (2 × 30 min), 75% acetone (2 × 1 h), 80% acetone (2 × 1 h), 95% acetone (2 × 1 h), and 100% acetone (2 × 2 h). Dehydrated samples were then embedded in Spurr’s resin as follows: Spurr: acetone (1:3 v/v) (3 × 2 h), Spurr: acetone (1:1 v/v) (3 × 2 h), Spurr: acetone (3:1 v/v) (2 × 2 h plus 1 × 3 h). All these steps were performed at 4°C.

After resin embedding, samples were left overnight at room temperature and newly included in 100% resin (2 × 3 h). Samples, placed on molds, were then incubated in pure resin at 60°C for 2–3 days in order to allow the polymerization. Finally, the semi-thin (0.7 μm) and ultrathin (50–70 nm) sections were prepared for light and electron microscopy, respectively. The ultrathin sections were contrasted in uranyl (2%) for 30 min, and in lead citrate for 12 min. Ultrathin sample assembling was carried out on copper grids of 100 and 200 mesh and examined by transmission electron microscopy (TEM), using a JEOL JEM-1010 transmission electron microscope (at 100 kV) (Peabody, MA, USA) equipped with a CCD Orius-Digital Montage Plug-in camera (Gatan Inc., Gatan, CA, USA) and Gatan Digital Micrograph software (Gatan Inc.).

### Mitochondrial membrane potential (ΔΨm)

Mitochondrial membrane potential was measured using the fluorochrome 5,5’,6,6’-tetrachloro-1,1’,3,3’-tetraethylbenzimidazolylcarbocyanine iodide (JC-1; Invitrogen, Molecular Probes, Renfrewshire, UK) as previously reported by Díaz-Tielas et al. [10]. After 7 and 14 days, RA-treated and untreated *Arabidopsis* roots were permeabilized in 5% DMSO for 1 h, and then incubated in 10 mg mL^-1^ JC-1 solution in the dark, for 30 min. A positive control, treated with 0.5 mg mL^-1^ valinomycin (Invitrogen, Molecular Probes), a mitochondrial membrane depolarizer, was also included. After incubation, root samples were visualized and photographed using a Leica TCS SP5 confocal microscope (Wetzlar, Germany) [equipped with three spectral detectors with a resolution of 8.192 X 8.192 and FRAP, FLIP, FRET, 3D and Dye Finder software (Leica Microsystems, Wetzlar, Germany)] with an excitation laser of 488 nm and an emission of 535 and 590 nm for the green fluorescence of the JC-1 monomers and for the red fluorescence of the J-aggregates, respectively Red and green fluorescence percentage were further quantified using the Image-Pro plus software (Media Cybernetics). For each image acquisition, root tissue was selected and the area of root surface, stained by red and green fluorescence, was quantified considering their sum as 100%. Colors different from red and green were excluded from the measurements.

### Trypan blue staining

Arabidopsis root cell death was evaluated as previously described by Díaz-Tielas et al. [10]. Roots of 40 seedlings untreated and treated with 175 μM RA, for 7 and 14 days, were soaked in an aqueous solution of Trypan blue (0.5% w/v) and incubated in the dark for 5 min. After incubation, roots were carefully washed with phosphate-buffered saline (PBS) pH 7.4 and visualized under a Nikon Eclipse 800 light microscope equipped with a Nikon DS-U2 camera. Root cells characterized by blue stain indicate the presence of cell death.

### Nuclear staining with DAPI and acridine orange/ ethidium bromide

Apoptotic nuclear staining was carried out using the ready-to-use DAPI kit NucBlue Fixed Cell Stain (Molecular Probes by Life Technologies). Untreated and RA-treated roots, for 7 and 14 days, were fixed with Image-IT Fix-Perm kit (Molecular Probes by Life Technologies) as detailed in the package insert. Epifluorescence microscopy (Olympus BX53) was used to observe the apoptotic features of nuclear condensation. Since DAPI staining allows only the identification of nuclear alteration, the presence of dead cells was further confirmed through the acridine orange/ ethidium bromide double staining, which allows the identification of both apoptotic and necrotic cells [32].

In particular, control and 175 μM RA-treated Arabidopsis root tips, for 7 and 14 days, were cut and rinsed in a lysis buffer composed by NaCl (2.5 M), Na_2_EDTA (100 mM), Tris base (10 mM), TritonX (1% v/v) and DMSO (1% v/v). They were then incubated for 10 min, homogenized and centrifuged (200 *g* for 10 min at 4°C). The supernatant was discarded, the pellet was washed thrice with phosphate buffer (pH 7.0) and then suspended in 25 μL of dye solution composed by 100 μg mL^-1^ of acridine orange and 100 μg mL^-1^ ethidium bromide prepared in PBS. Successively, 10 μL of the stained cells were mounted on a microscope slide and observed using an epifluorescence microscope (Olympus BX53) with different filter combinations. The number of apoptotic and necrotic cells was evaluated on three independent replicates where each replicate was represented by ten roots.

### Extraction, identification, and quantification of metabolites

Extraction, identification, and quantification of metabolites from RA- treated and untreated roots, for 7 and 14 days, were performed using a Thermo Fisher gas chromatograph apparatus (Trace 1310) equipped with a single quadrupole mass spectrometer (ISQ LT). The capillary column was a TG-5MS 30 m×0.25 mm×0.25 μm, the gas carrier was helium. Sample extraction and derivatization were performed according to Araniti et al. [13].

The derivatized extract was injected into a TG-5MS capillary column with injector and source settled at 250°C and 260°C temperature, respectively. One microliter of sample was injected in splitless mode with a flow of 1 mL min^-1^ using the following programmed temperature: isothermal 5 min at 70°C followed by a 5°C/ min ramp to 350°C and a final 5 min heating at 330°C. Mass spectra were recorded in electronic impact (EI) mode at 70 eV, scanning at 45–500 m/z range.

The extracted metabolites were identified via comparing every retention time index-specific mass with reference spectra in mass spectral libraries (NIST 2005, Wiley 7.0 etc.). Relative metabolites quantification was based on internal standard (20 μg mL^-1^ ribitol) added during the extraction process.

### Experimental design and statistical analysis

Experiments were carried out using a completely randomized design with 4 replications. Data were checked for normality through the Kolmogorov-Smirnov test and then tested for homogeneity of variances with the Levene’s test. Differences among group means were statistically evaluated by analysis of variance followed by Least Significant Difference tests (LSD) in the case of homoscedastic data, and by Tamhane’s T2 test in the case of heteroscedastic data (*P* ≤ 0.05).

All the statistical analyses were conducted using SPSS *ver*. 6.1 software (Insightful Corporation, USA). Root morphological parameters in response to RA-increasing doses were evaluated by a nonlinear regression model using a log-logistic function in order to identify the ED_50_ as reported by Araniti et al. [9].

Metabolomic data were analyzed using the software Metaboanalyst 3.0 [33]. Data, expressed as metabolite concentrations, were checked for integrity and missing values were replaced with a small positive value. Successively, data were normalized by the pre-added internal standard (ribitol), transformed through “Log normalization” and scaled through Pareto-Scaling. Data were then classified through a Principal Component Analysis (PCA) and metabolite variations were presented as heatmap. Differences between treatments were considered significant with *P ≤* 0.05 (Student’s *t*-test). Finally, the identification and visualization of the affected metabolic pathways were performed using a proof-of-knowledge based MetPA (Metabolic Pathway Analysis) with Metaboanalyst as previously reported by Araniti et al. [13].

## Results

### Root morphology

**Fig 1:**
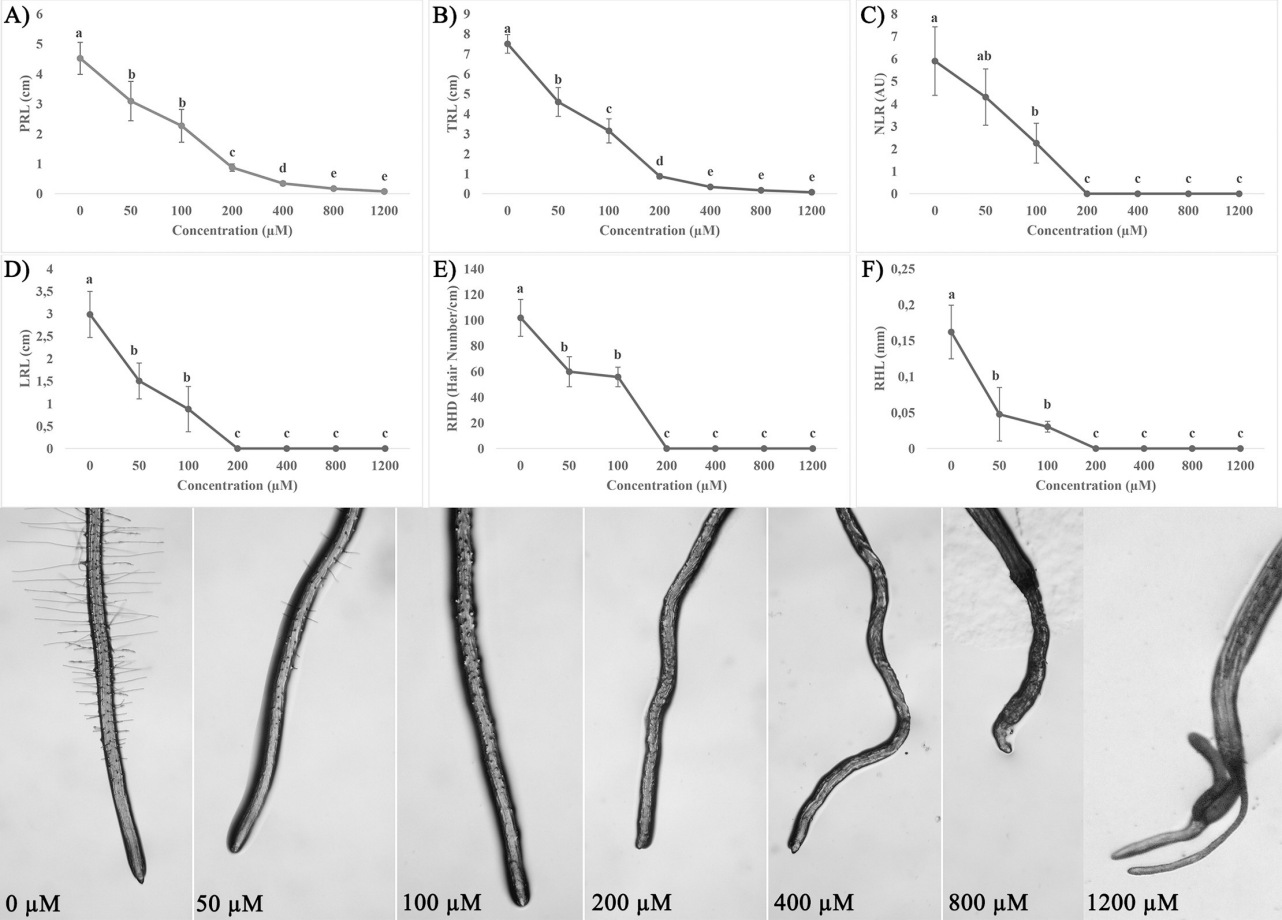
Effects of rosmarinic acid on root morphology of *A*. *thaliana*. A) Primary Root Length (PRL), B) Total Root Length (TRL), C) Number of Lateral Roots (NLR), D) Lateral Roots Length (LRL), E) Root Hair Length (RHL), and F) Root Hair Density (RHD) of *Arabidopsis* seedlings treated with different concentrations (0–1200 μM) of RA, for 14 days. On the bottom the effects of different RA concentrations on root anatomy are reported. Data are expressed as percentage of the control. Different letters indicate significant differences among treatments at *P* ≤ 0.05. N = 4.

Rosmarinic acid significantly affected root morphology causing an inhibition of both total and primary root length (TRL and PRL) with 71 μM and 175 μM ED_50_ values, respectively (Fig 1A and 1B). All the other root morphological traits were also affected by RA-treatments. In particular, RA significantly reduced the lateral roots number (NRL) with an ED_50_ value by about 53 μM, reaching the complete inhibition at higher RA concentrations (Fig 1C). The lateral root length (LRL) was also significantly affected already at the lowest concentration with a ED_50_ value close to 100 μM (Fig 1D; Table 1), as well as the root hair length (RHL) and density (RHD) (Fig 1E and 1F). Interestingly, NLR, RLR, RHD and RHL were completely inhibited by RA at the same tested concentration, with a LCIC (Lowest Complete Inhibitory Concentration) value below 200 μM RA, which is very low for a natural compound. Moreover, RA caused a change on root hair anatomy, showing, at 50 μM, a high number of dichotomic root hairs in treated plants (S1 Fig). After 14 days of treatment, seedlings continued to survive but plant development was extremely reduced and seedlings were characterized by high malformations (data not shown).

**Table 1:** Effects of rosmarinic acid on root morphology of *A*. *thaliana*. ED_50_ (μM) values for primary root length (PRL), total root length (TRL); number of lateral roots (NLR), lateral roots length (LRL), root hair length (RHL), and root hair density (RHD) of *Arabidopsis* seedlings treated for 14 days with different concentrations (0–1200 μM) of RA.

Root parameters	ED_50_ (μM)
**PRL (cm)**	175 (± 6.54)
**TRL (cm)**	71.13 (± 3.51)
**LRL (cm)**	99.07 (± 13.12)
**NRL (AU)**	52.76 (± 4.35)
**RHL (mm)**	19.42 (± 8.7)
**RHD (Hair number/cm)**	83.30 (± 12.68)

Data were estimated by the log-logistic equations in response to increasing doses of RA. Data from Fig 1A–1F. Values within the brackets indicate the standard deviation (N = 4). All the dose-response curves pointed out a significance level of *P* < 0.001.

The experiments carried out with NaN_3_ (positive control), a known SOD and CAT inhibitor, pointed out a similar trend of phytotoxicity and a phenotype similar to RA-treated roots. In particular, all the root morphology traits (Eg. PRL, LRN, RHD) were negatively affected by NaN_3_ treatments, and treated roots showed anatomical malformations (eg.: dichotomic and bulbous root hairs) similar to those induced by RA treatment (S2 Fig).

### Root cell ultrastructure

**Fig 2:**
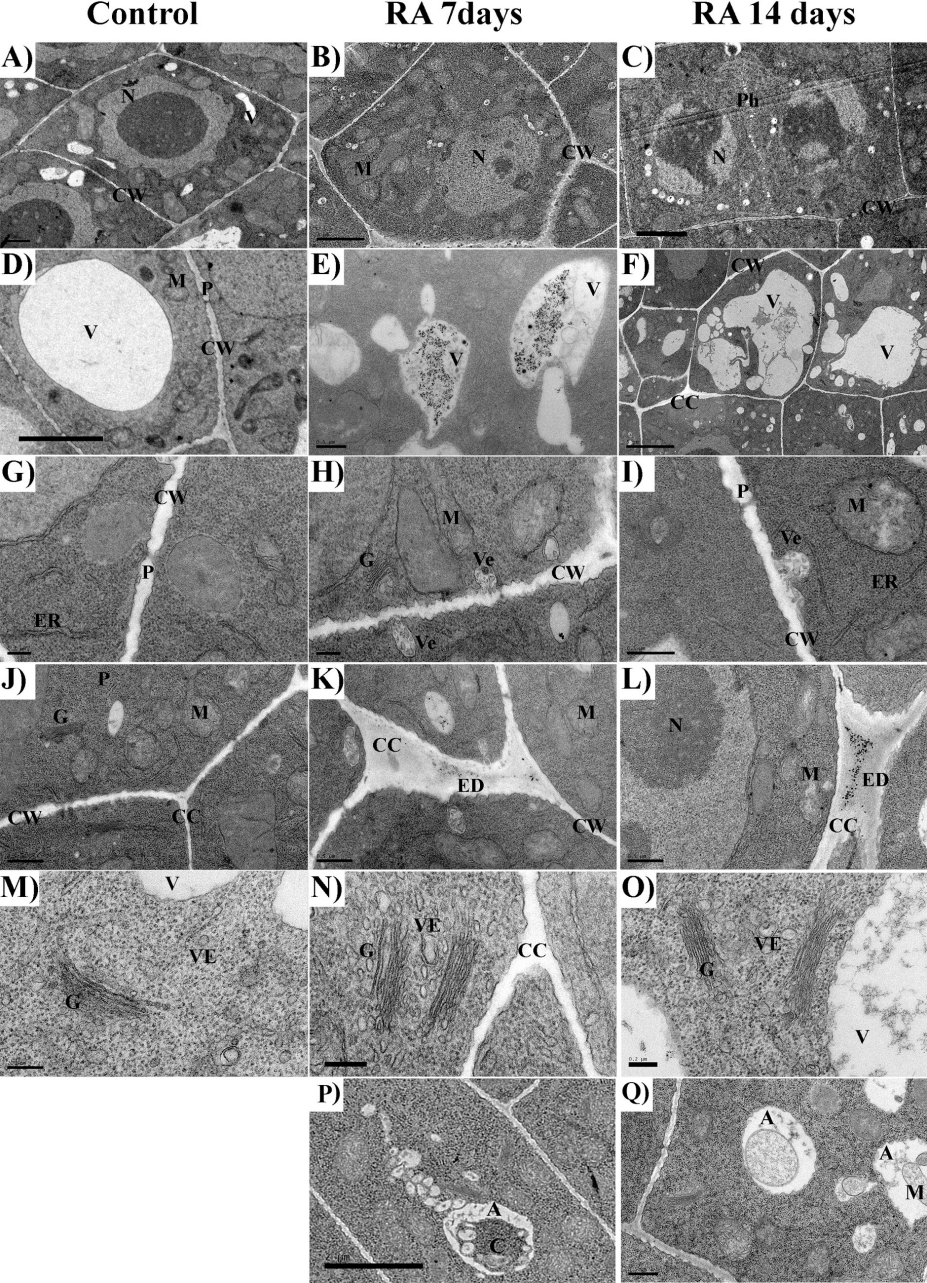
TEM images of rosmarinic acid-treated and untreated Arabidopsis meristems. TEM images of the apical meristem of control (A, D, G, J, M), and 7 (B, E, H, K, N) and 14 days (C, F, I, L, O) RA-treated (175 μM) Arabidopsis roots: A) control cell of the stele, it should be noted the smooth contour of the nuclei; B) 7 days treated cell of the stele, it should be noted the wavy contour of the nucleus; C) 14 days treated cell of the stele, it should be noted the irregular shape of the nuclei and the presence of fragmented chromatin; D) small vacuole from untreated cell, it should be noted the smooth tonoplast and few fibrillary content; E) small vacuole from 7 days treated cell, it should be noted the presence of a blurry tonoplast and a central accumulation of electrodense material and fine granular material throughout the vacuole volume; F) huge vacuole from 14 days treated cell, it should be noted the classical lytic shape of the vacuole ant the irregular membrane-bound structures containing dense granulose material; G) cell wall of untreated cell with plasmodesmata; H,I) cell walls from 7 and 14 days treated cell with accumulation of electron dense droplets and numerous vesicles in the plasma membrane/cell wall interspace; J) cell corner from untreated cell; K,L) swollen cell corners from 7 and 14 days treated cells with accumulation of electron dense deposits; M) Golgi apparatus from untreated cell; N,O) Golgi apparatus from 7 and 14 days treated cells; should be noted the high production of vesicles; P) single membrane autophagosome in 7 days treated roots, Q) double membrane autophagosome in 14 days treated roots. Nucleus (N), vacuole (V), cell wall (CW), mitochondria (M), phragmoplast (Ph), electrodense deposits (ED), cell corner (CC), plasmodesmata (P), endoplasmic reticulum (ER), Golgi apparatus (G), vesicles (Ve), tonoplast (T), autophagosome (A), cytoplasm (C), mitochondria (M).

**Fig 3:**
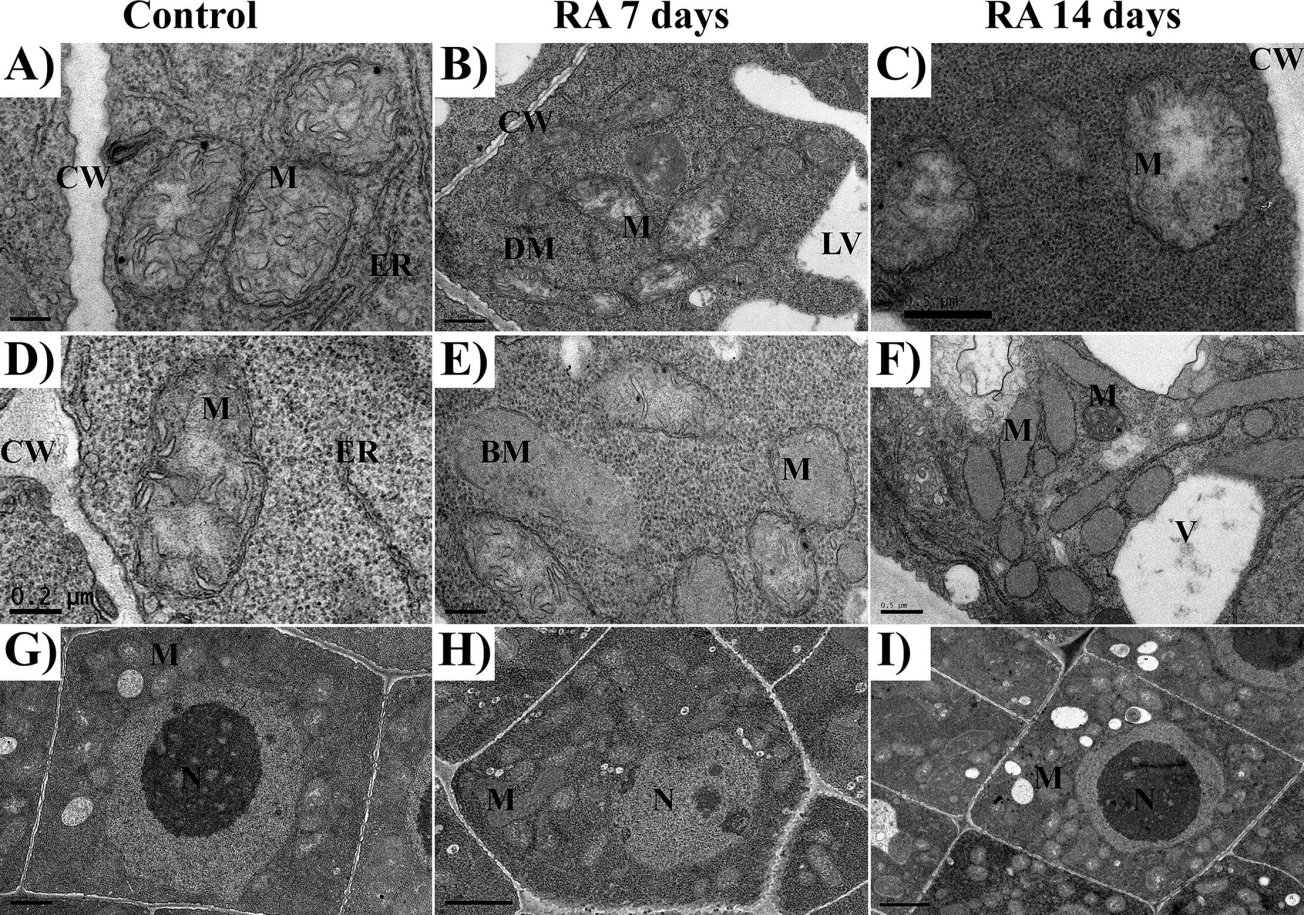
TEM images of mitochondria in rosmarinic acid-treated and untreated Arabidopsis meristems. TEM images of the apical meristem of control (A, D), and 7 (B, E) and 14 days (C, F) RA-treated (175 μM) Arabidopsis roots: A, D) mitochondria from control cells; B, C) mitochondria from 7 and 14 days treated cells, it should be noted the presence of translucent stroma; E) mitochondria from 7 days treated cells, it should be noted the presence of broken mitochondria and the reduced number of cristae; F) mitochondria from 14 days treated cells characterized by a strong reduction in the number of cristae; G-I) Control cell (G), 7 days (H) and 14 days (I) RA-treated cells, should be noted the high increment in mitochondria number in treated cells compared to control cell. Cell wall (CW), lytic vacuole (LV), mitochondria (M), dividing mitochondria (DM); broken mitochondria (BM); endoplasmic reticulum (ER), Golgi apparatus (G).

The ultrastructural analysis pointed out that RA strongly altered root cell structure and organization in treated roots, inducing a plethora of modifications. Different organelles, such as vacuoles, nuclei, mitochondria or Golgi system were affected by RA treatment (Figs 2 and 3 and Table 2).

**Table 2:** Quantification of changes detected in the cell structure and the organelles of untreated (0 μM) and RA-treated (175 μM) Arabidopsis roots.

Observations	7 days RA-treatment		14 days RA-treatment	
	0 μM	175 μM	0 μM	175 μM
**Cell wall thickness**	0.12 ± 0.014	0.20 ± 0.089*	0.09 ± 0.020	0.18 ± 0.059**
**Density of Plasmodesmata**	2.85 ± 0.94	2.32 ± 0.66	1.98 ± 0.57	2.16 ±0.53
**Density of abnormal nuclei (condensed chromatin)**	0.013 ± 0.005	0.067± 0.045	0.003 ± 0.005	0.047 ± 0.015***
**Density of cell wall deposits**	0.035 ± 0.02	0.192 ± 0.026***	0.047 ± 0.028	0.162 ± 0.03***
**Density of autolytic vacuoles**	0.027 ± 0.01	0.087 ± 0.02*	0.032 ± 0.017	0.13 ± 0.053*
**Density of altered vacuoles with deposits**	0.12 ± 0.09	0.42 ± 0.04*	0.18 ± 0.14	0.71 ± 0.08*
**Density of autophagosomes**	0.013 ± 0.005	0.09 ± 0.03*	0.015 ± 0.012	0.1 ± 0.026***
**Density of mitochondria**	0.665 ± 0.21	1.31 ± 0.28***	0.703 ± 0.172	1.404 ± 0.303*
**Density of altered mitochondria**	0.044 ± 0.05	0.53 ± 0.15***	0.033 ± 0.043	0.0867 ± 0.261***

Ultrastructure measurements were expressed as: cell wall thickness (μm); Density of plasmodesmata (n° of plasmodesmata / μm of cell wall); Density of abnormal nuclei (n° of aberrant nuclei / 100 cells); Density of cell wall deposits (n° of cell wall deposits / 100 cells); Density of autolytic vacuoles (n° of autolytic vacuoles / 100 cells); Density of altered vacuoles with deposits (n° of altered vacuoles / 100 cells); Density of autophagosomes (n° of autophagosomes / 100 cells); Density of mitochondria (n° of mitochondria / 100 cells); Density of broken/altered mitochondria (n° of broken and altered mitochondria /100 cells). Data were expressed as mean ± SD. Differences between treatments were statistically analyzed through t-test with *P* ≤ 0.05.

* = (*P* ≤ 0.05)

** = (*P* ≤ 0.01)

*** = (*P* ≤ 0.001).

In RA-treated cells, the tonoplast of most of the vacuoles was blurry and not easily recognizable (Fig 2E and 2F) compared to the control cells, where the few vacuoles were characterized by a small dimension, containing sparse fibrillar material surrounded by a highly recognizable tonoplast (Fig 2D). Moreover, RA-treated cells showed several inclusions, especially aggregations of granular precipitates located centrally or peripherally in the vacuole (Fig 2E). In addition, irregular membrane-bound structures, containing dense granulose material within vacuoles, were commonly found (Fig 2F). In particular, such vacuole alterations were 3.4 folds (7 days) and 4 folds (14 days) higher than in control cells (Table 2).

Although nuclei of RA-treated roots did not significantly differed from untreated ones, except for their wavy appearance, after 7 days of treatment (Fig 2B), a high number of nuclei with irregular shape and condensed chromatin (14 folds higher than control) were observed after 14 days RA-treatment (Fig 2C and Table 2).

No significant differences were observed in the Golgi complexes morphology, which was similar in both control and treated cells. However, the surrounding of the Golgi apparatus in RA-treated cells, in the interspace between cell wall and plasma membrane, was characterized by a high number of vesicles, as a probable signal of its increased secretory activity (Fig 2H and 2I). Furthermore, cell corners were extremely affected by RA-treatment (Fig 2K and 2L) compared to the control (Fig 2J), as they appeared swollen, with an abnormal shape and rich in electrodense deposits, which were 5.5 folds (7 days) and 3.4 folds higher than the control (Table 2). Moreover, RA-treatment significantly increase the thickness of primary cell walls (0.6 and 0.84 folds, after 7 and 14 days, respectively), whereas the number of plasmodesmata was not affected (Fig 2G and Table 2).

Regarding to mitochondria number and morphology, the control cells showed pleomorphic mitochondria delimited by a discernible double membrane, containing numerous cristae in a dense stroma (Fig 3A and 3D). By contrast, in RA-treated cells, a higher number of mitochondria [2 folds higher than control after both 7 and 14 days of treatment (Table 2)], characterized by different shapes, from oval to spherical, was observed. Some of them were broken while others progressively lost their cristae being characterized by a translucent to transparent stroma (Fig 3B, 3C and 3E). However, this effect appeared to be gradual, since damaged and intact mitochondria co-existed after 7 days of treatment (Fig 3). Conversely, after 14 days, mitochondria without cristae, with translucent and/or almost transparent stroma, containing granular material surrounded by double membrane envelopes, were observed (Fig 3B and 3F). In particular, the density of altered mitochondria was 12 (7 days) and 26 (14 days) folds higher than the control (Table 2).

Finally, a higher number of autophagosome in RA-treated roots (7.2 and 6.7 folds higher than the control, after 7 and 14 days, respectively), and an increment of autolytic vacuoles (3.2 and 4 folds higher than the control, after 7 and 14 days, respectively) were observed (Fig 2P and 2Q and Table 2).

### Mitochondrial membrane potential (ΔΨm)

**Fig 4:**
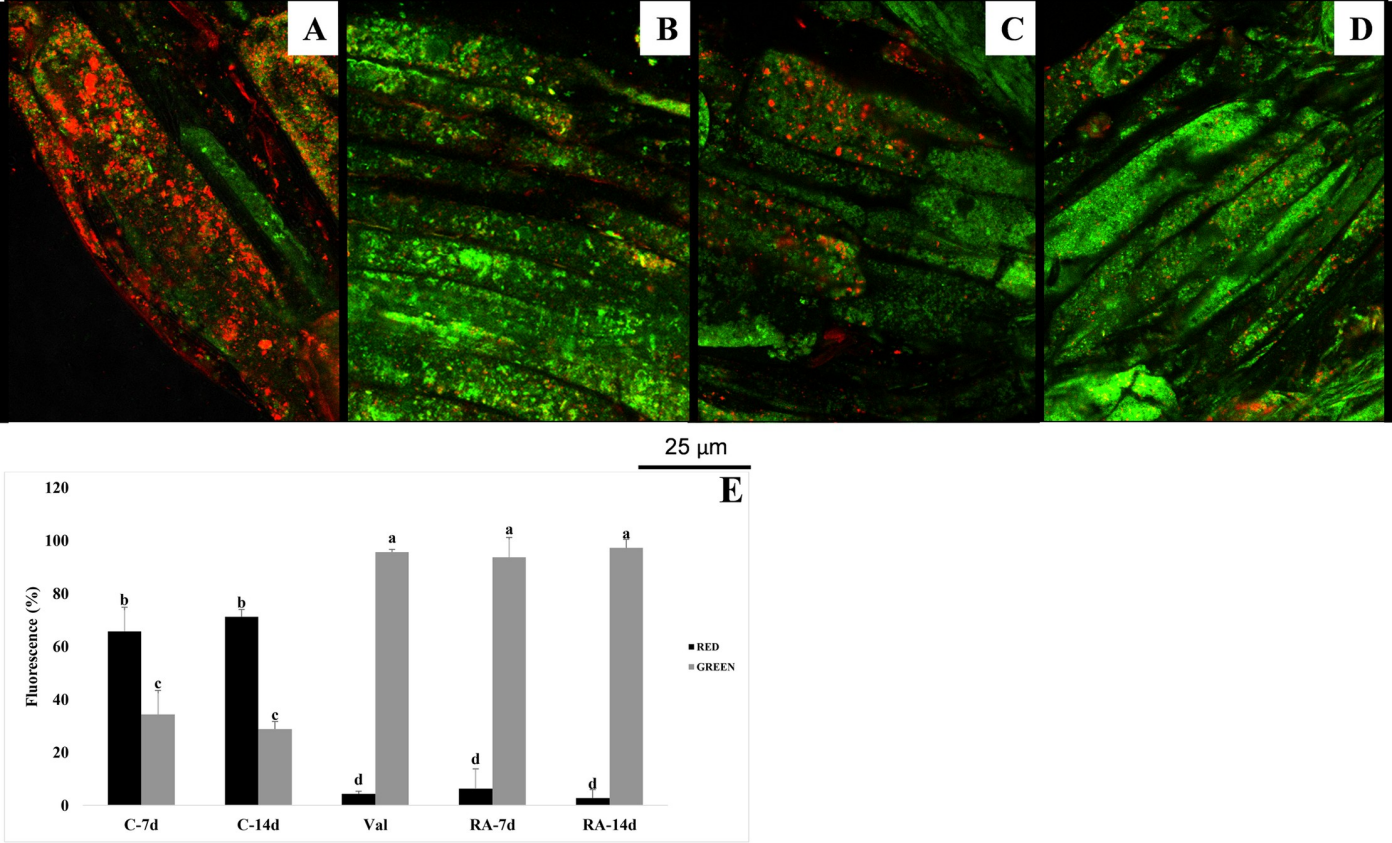
Effects of rosmarinic acid on mitochondrial membrane potential. Mitochondrial membrane potential (Δ*Ψm*) in meristematic root cells of Arabidopsis thaliana treated with 175 μM RA and stained with the fluorochrome JC-1. Negative control (A): huge number of mitochondria red-stained due to the formation of JC-1 aggregates thanks to the highly negative Δ*Ψm*. Positive control (valinomicyn) (B) and RA treated cells (C-D): green fluorescence due to the cytoplasmic presence of the JC-1 monomers due to mitochondrial membrane depolarization. Percentage of red and green fluorescence in 7 and 14 days old control roots (C-7d and C-14d), in valinomicyn treated roots (Val) and in 7 and 14 days old RA-treated roots (RA-7d and RA-14d) (E). N = 4.

RA caused a strong inhibitory effect on mitochondrial membrane potential (ΔΨ_m_) after both 7 and 14 days of treatment (Fig 4). Control roots showed a high number of red-stained mitochondria due to JC-1 fluorochrome penetration into the membrane of respiring mitochondria, where it formed aggregates (Fig 4A and 4E). This penetration generally happens only in healthy mitochondria characterized by a high ΔΨ_m_.

Conversely, in depolarized membranes (low ΔΨ_m_) of RA-treated cells, the fluorochrome was not able to penetrate and aggregate inside the mitochondria, remaining in the cytoplasm as monomeric green fluorescent form. This effect was similar to that induced by the positive control valinomycin, a well-known inducer of cell membrane depolarization (Fig 4B–4E).

### H_2_O_2_ and O_2_^−^ localization and lipid peroxidation

**Fig 5:**
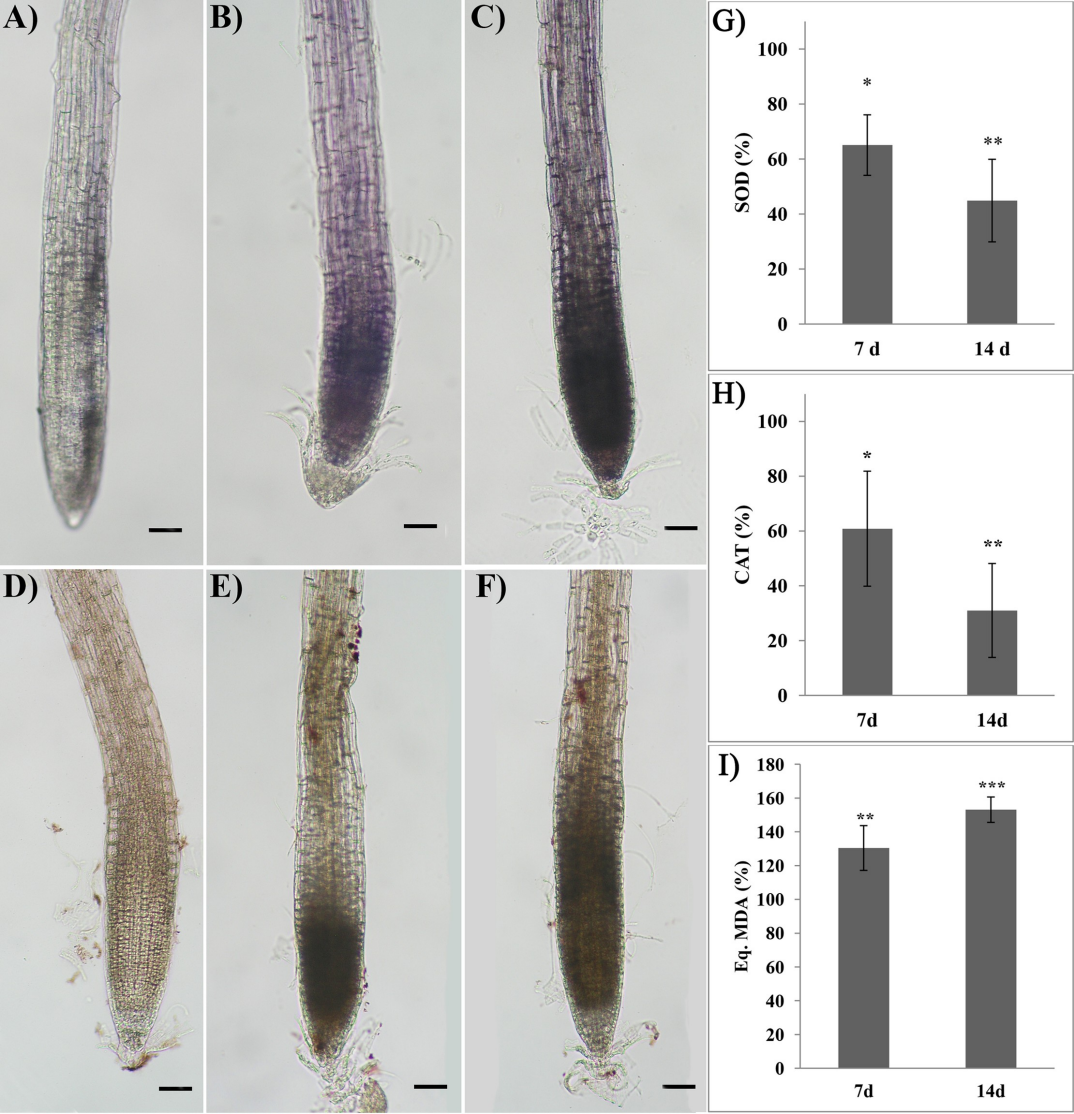
Oxidative stress induced by rosmarinic acid treatment and ROS scavenging activity. *In situ* superoxide (A-C) and hydrogen peroxide (D-F) localization in roots of Arabidopsis treated with 175 μM RA for 7 and 14 days. Control (A, D), 7 days (B, E) and 14 days (C, F) treatments. G) Superoxide dismutase, H) Catalase and I) Lipid peroxidation in roots of Arabidopsis treated with 175 μM RA for 7 and 14 days. Image magnification 12X. Black bars = 200 μm. Data are given as percentage compared to the control. N = 4. Asterisks indicate significant differences compared to the control: * (*P* ≤ 0.05), ** (*P* ≤ 0.01), *** (*P* ≤ 0.001); data were analyzed through t-test with *P* ≤ 0.05.

After 7 and 14 days of treatment, RA-treated roots pointed out a strong production of both measured ROS, O_2_^–^ and H_2_O_2_ (Fig 5). *In situ* O_2_^-^ and H_2_O_2_ localization revealed that oxidative burst started at the root distal part in cell division and moved towards the elongation zone as far as the treatment proceeded. Treated roots showed a significant inhibition of both SOD and CAT activities (Fig 5G and 5H). In particular, SOD activity was reduced by 35% and 55% after 7 and 14 days of treatment, respectively (Fig 5G). A similar trend was observed in CAT activity, which was reduced by 40% after 7 days, reaching 69% of inhibition at the end of the experiment (Fig 5H).

The *in-vitro* experiments confirmed that RA directly inhibited SOD and CAT enzymes reducing their activity by 29% and 64%, respectively (S3 Fig).

On the contrary, lipid peroxidation was significantly increased by the RA-treatment pointing out an increment of 30% and 53% after 7 and 14 days, respectively (Fig 5I).

### Cell death

**Fig 6:**
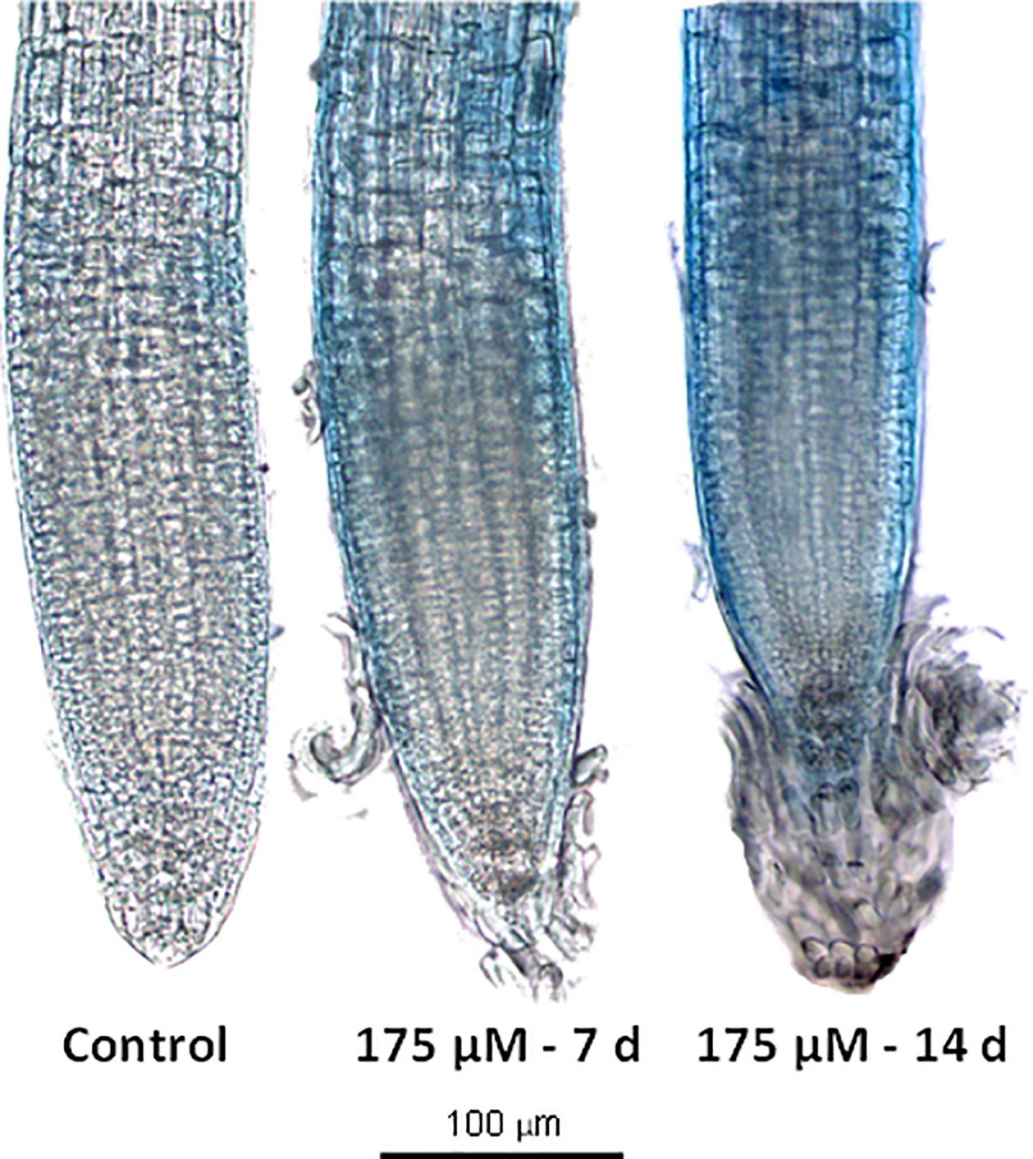
Cell death evaluation on rosmarinic acid-treated *A*. *thaliana* root meristem. Trypan blue staining of Arabidopsis roots after 7 and 14 d of growth with 0 and 175 μM RA. N = 4.

RA caused cell death in Arabidopsis root meristems after both 7 and 14 days of treatment. As reported in Fig 6, control roots did not show any sign of staining, indicating that cells were viable. Conversely, after 7 and 14 days, RA-treated roots showed a significant increment of the staining intensity denoting dead cells presence, especially in both the meristematic and differentiation zones (Fig 6).

**Fig 7:**
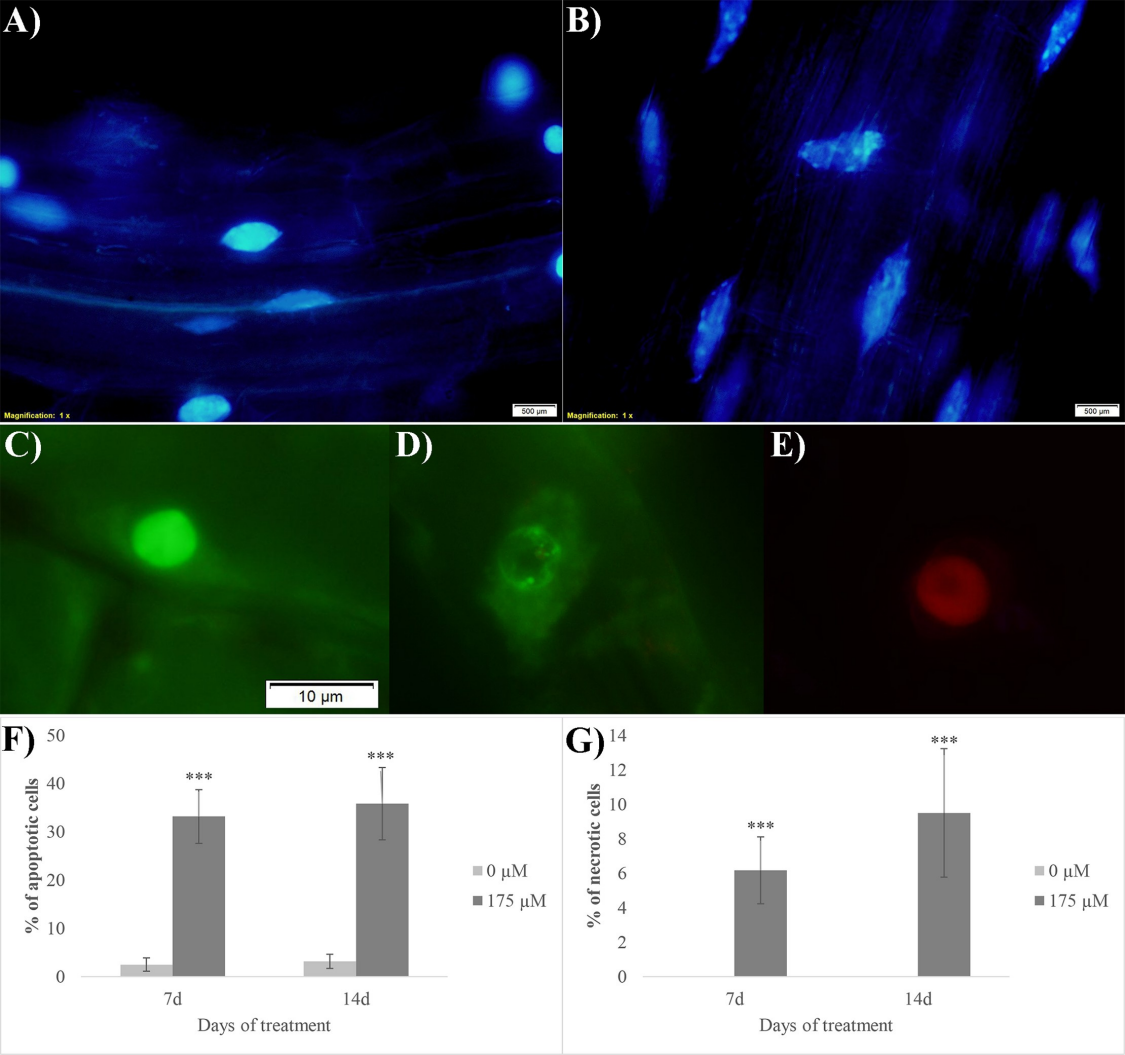
Cell death evaluation on rosmarinic acid-treated *A*. *thaliana* root meristem. Images of cells double stained with DAPI (A-B) and acridine orange/ethidium bromide (C-E): A) nuclei of the control cells stained with DAPI, B) nuclei of the RA-treated cells stained with DAPI with fragmented chromatin, C) viable nuclei, D) early apoptotic cells, E) necrotic cells. Similar images were observed in both 7 days and 14 days RA-treated roots. F) % of apoptotic cells (evaluated as number of apoptotic cells/100 cells); G) % of necrotic cells (evaluated as number of necrotic cells/100 cells). Asterisks indicate significant differences compared to the control: * (*P* ≤ 0.05), ** (*P* ≤ 0.01), *** (*P* ≤ 0.001); data were analyzed through t-test with P ≤ 0.05. N = 3.

The DAPI nuclear staining evidenced clear signs of apoptosis in RA-treated root tips (Fig 7A and 7B). In fact, in the control cells, nuclei (Fig 7A) were characterized by a blue and uniform color, indicating that chromatin was preserving its integrity, whereas in the RA-treated nuclei a clear fragmentation of chromatin was observed (Fig 7B). Furthermore, the acridine orange/ethidium bromide double staining pointed out the presence of both early apoptotic and necrotic cells (Fig 7C and 7E). In particular, nuclei of the viable control cells were characterized by uniform bright green (Fig 7C), while early apoptotic cells showed green nuclei with intact membrane accompanied by a visible perinuclear chromatin condensation (visible as bright green patches or fragments) (Fig 7D). Finally, necrotic cells showed uniformly orange nuclei with organized structure (Fig 7E). After 7 and 14 days of growth, the control roots showed 2.5% and 3.1% of apoptotic cells, respectively, while these numbers raised up to 33% and 36% in RA-treated roots, respectively (Fig 7F). Furthermore, no sign of necrosis was observed in control roots, whereas its incidence was by 6% and 9.5% in 7 and 14 days RA-treated roots, respectively (Fig 7G).

### Metabolomic analysis

The GC-MS analysis carried out on RA-treated roots for 14 days allowed to identify and quantify 57 metabolites, including 19 amino acids, 1 acetylated amino acid, 18 organic acids, 9 sugars, 2 sugar alcohols, 4 fatty acids and 4 amines (Table 3).

**Table 3:** Effects of rosmarinic acid treatment on metabolites content. Chemical compounds isolated and quantified trough GC-MS in Arabidopsis roots treated for 14 days with 0 or 175 μM rosmarinic acid.

Compound	0 μM	175 μM	[Variation]	*P* value	Class
	ng g^-1^ FW		%		
ß-Alanine	5.27	4.57	↓ 13.3	0.00028333	Amino acids
Citrulline	86.02	47.64	**//**	NS	
GABA	38.27	16.49	↓ 56.9	0.04231	
Glutamic acid	298.52	143.08	↓ 52.1	0.016895	
Glycine	61.29	26.10	↓ 57.4	0.00012255	
L-Alanine	16.38	10.15	**//**	NS	
L-Asparagine	417.16	299.24	↓ 28.3	0.013355	
L-Aspartic acid	194.09	82.10	↓ 57.7	0.013916	
L-Glutamine	585.82	349.21	**//**	NS	
L-Lysine	7.95	7.63	↓ 3.9	7.4508e-06	
L-Ornithine	165.18	139.65	↓ 15.5	0.012991	
L-Proline	55.15	50.61	↓ 8.2	4.7145e-07	
L-Threonine	63.95	35.13	**//**	NS	
L-Valine	3.24	1.55	**//**	NS	
N-Acetyl-D-glucosamine	74.68	34.02	↓ 54.4	0.045316	
Norvaline	1.04	2.05	↑ 97.9	1.0416e-05	
Putrescine	20.36	41.27	↑ 102.7	1.1758e-05	
Serine	408.81	166.97	↓ 59.2	0.0071781	
Thymidine	5.02	3.27	**//**	NS	
N-α-acetyllysine	7.11	14.63	↑ 105.6	0.00019011	Acetylated amino acids
Oxoglutarate	20.97	6.51	↓ 69.0	0.0014108	Organic acids
Benzoic acid	8.14	11.01	**//**	NS	
Citric acid	8.68	3.73	↓ 57.1	0.006881	
Gluconate	19.69	18.31	↓ 7.0	0.00037923	
Glucuronate	59.13	55.82	↓ 5.6	0.00022341	
Fumaric acid	178.20	61.85	↓ 65.3	0.00015616	
Glyceric acid	5.19	4.85	↓ 6.4	0.00023962	
Glycolate	8.29	2.90	↓ 65.0	0.0033541	
Lactic acid	83.75	32.87	↓ 60.7	0.010511	
L-Malic acid	104.01	35.47	↓ 65.9	1.1167E-05	
Malonic acid	1.59	1.23	↓ 22.9	0.0080257	
Oxalic acid	10.45	3.90	**//**	NS	
Phosphoric acid	764.40	101.62	↓ 86.7	1.2699E-06	
Pyroglutamic acid	415.75	280.45	**//**	NS	
Pyruvic acid	1.87	0.67	↓ 64.3	4.0329E-05	
Sinapic acid	65.21	46.36	↓ 28.9	0.0032338	
Succinic acid	19.23	6.82	↓ 64.5	0.00022059	
Threonic acid	14.97	15.80	↑ 5.5	0.0012131	
D-Fructose	236.13	469.18	↑ 98.7	4.3808E-05	Sugars
D-Glucose	82.74	167.77	↑ 102.8	0.00015005	
D-Mannose	423.44	400.25	↓ 5.5	0.00041125	
Lactulose	71.67	49.49	↓ 30.9	0.0016069	
Maltose	13.64	42.03	↑ 208.1	7.9987E-06	
Mannobiose	12.87	13.18	↑ 2.5	4.0713E-05	
Sucrose	649.64	365.17	↓ 43.8	0.0070796	
Threose	64.21	21.54	↓ 66.5	0.0080599	
Turanose	155.27	82.33	**//**	NS	
Glycerol	6.46	4.68	**//**	NS	Sugar alcohol
Myoinositol	289.62	118.10	↓ 59.2	0.0037223	
Myristic acid	4.49	2.54	**//**	NS	Fatty acids
Octadecanoic acid	126.08	68.17	**//**	NS	
Palmitic acid	77.88	52.73	**//**	NS	
Adipic acid	4.13	2.92	**//**	NS	
Ethanolamine	4.60	2.17	**//**	NS	Amines
Hydroxylamine	107.29	61.04	**//**	NS	
Acetamide	4.09	2.15	**//**	NS	
Urea	15.52	1.56	↓ 90	2.9033E-07	

Important features selected by *t*-tests with threshold ≤ 0.05. NS: Not Significant features. ↑) metabolites significantly increased in treated plants; ↓) Metabolites significantly reduced in treated plants; **//**) Not significant variations. N = 4.

**Fig 8:**
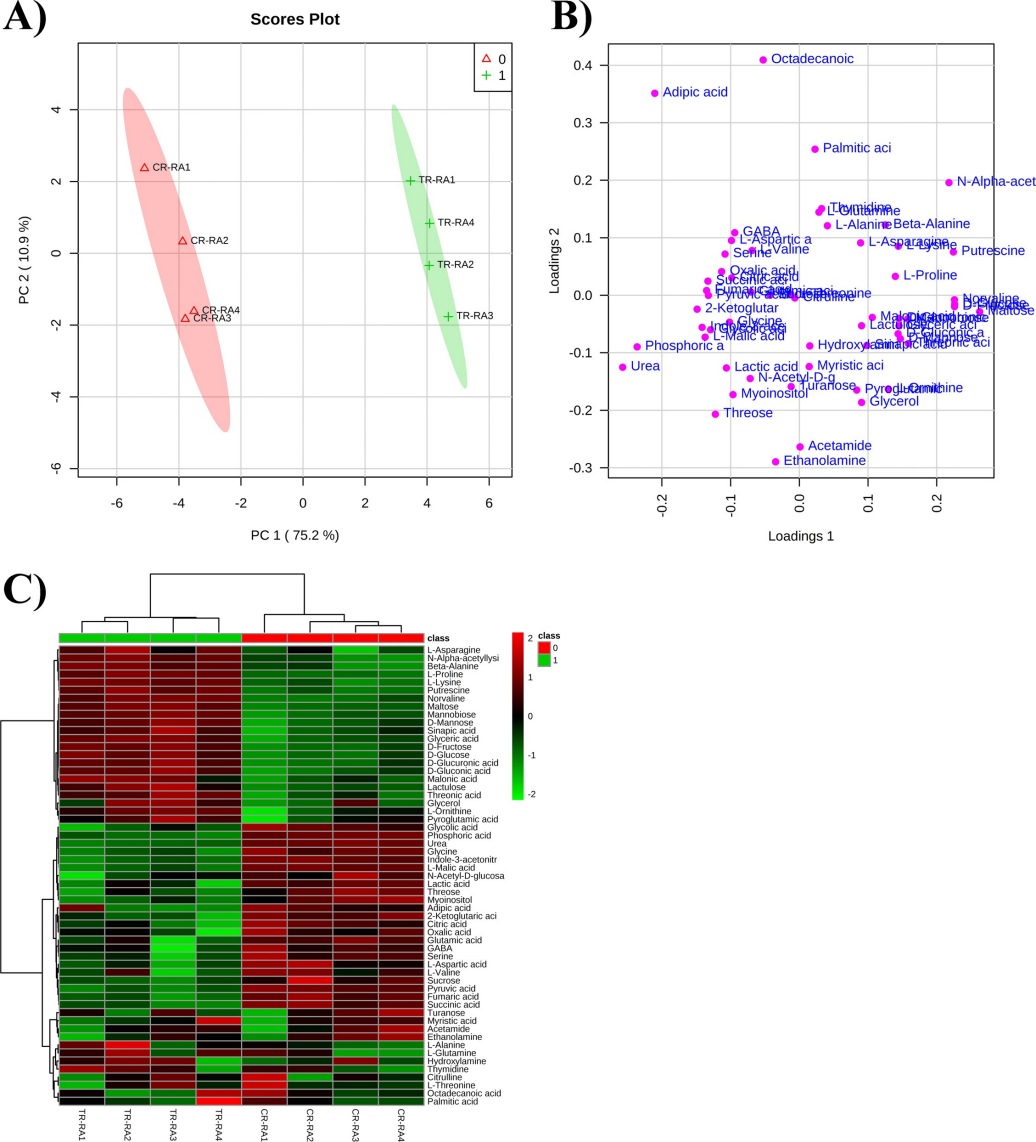
PCA analysis carried on the metabolite identified and quantified after rosmarinic acid treatment. Principal Component Analysis model scores A) and loading B) plot of metabolite profile of control plants (CR-RA1– CR-RA4, replicates of control samples) and plants exposed to RA (TR-RA1– TR-RA4, replicates of the treated samples). Both score and loading plots were generated using the PCs, PC1 *vs* PC2, with the explained variances reported in brackets; C) Overlay heat map of metabolite profiles in Arabidopsis roots exposed to RA in comparison with control roots. Heatmap is commonly used for unsupervised clustering. Agglomerative hierarchical clustering begins with each sample as separate cluster and then proceeds to combine them until all samples belong to one cluster. Each square represents the effect of RA on the amount of every metabolite using a false-color scale. Red or green regions indicate increase or decrease metabolite content, respectively.

The multivariate data analysis of the raw data, carried out through Principal Component Analysis (PCA), indicated that the metabolic profile of the control and treated roots was clearly separated, confirming that RA treatment was changing root metabolism (Fig 8A and 8B). The separation between control and treated roots was achieved using the principal components (PCs) PC1 *vs* PC2, which explained a total variance of 86.1%. The PC1 was the component characterized by the highest variance (75.2%), whereas PC2 was the component with the lowest variance (10.9%) in the subspace perpendicular to PC1 (Fig 8A).

The PCA loading plot showed that PC1 was largely characterized by the presence of urea and phosphoric acid as well as maltose, glucose, fructose and norvaline. On the other hand, PC2 was dominated by octadecanoic acid, adipic acid, acetamide and ethanolamine (Fig 8B). Finally, the clusterization of the control and treated roots and the variation in concentration of each single metabolite has been reported in the heatmap (Fig 8C).

Data were then analyzed through the *t*-test analysis (*P* ≤ 0.05) to identify statistically significant differences in metabolite concentrations between control and treated roots. The univariate analysis showed that 39 out of 57 identified metabolites were significantly affected by RA-treatment (Table 3). These metabolites were further analyzed through MetPa, a metaboanalyst module, which allowed the identification of the most relevant pathways affected by RA treatment, combining the results of the powerful pathway enrichment analysis with those of pathway topology. In particular, the metabolic pathway analysis revealed that 13 pathways were significantly affected by RA-treatment (S4 Fig). Among them, ß-alanine, alanine, glutamate and aspartate metabolism as well as glycine, serine, threonine metabolism and the TCA cycle were the most impacted (S4 Fig).

**Fig 9:**
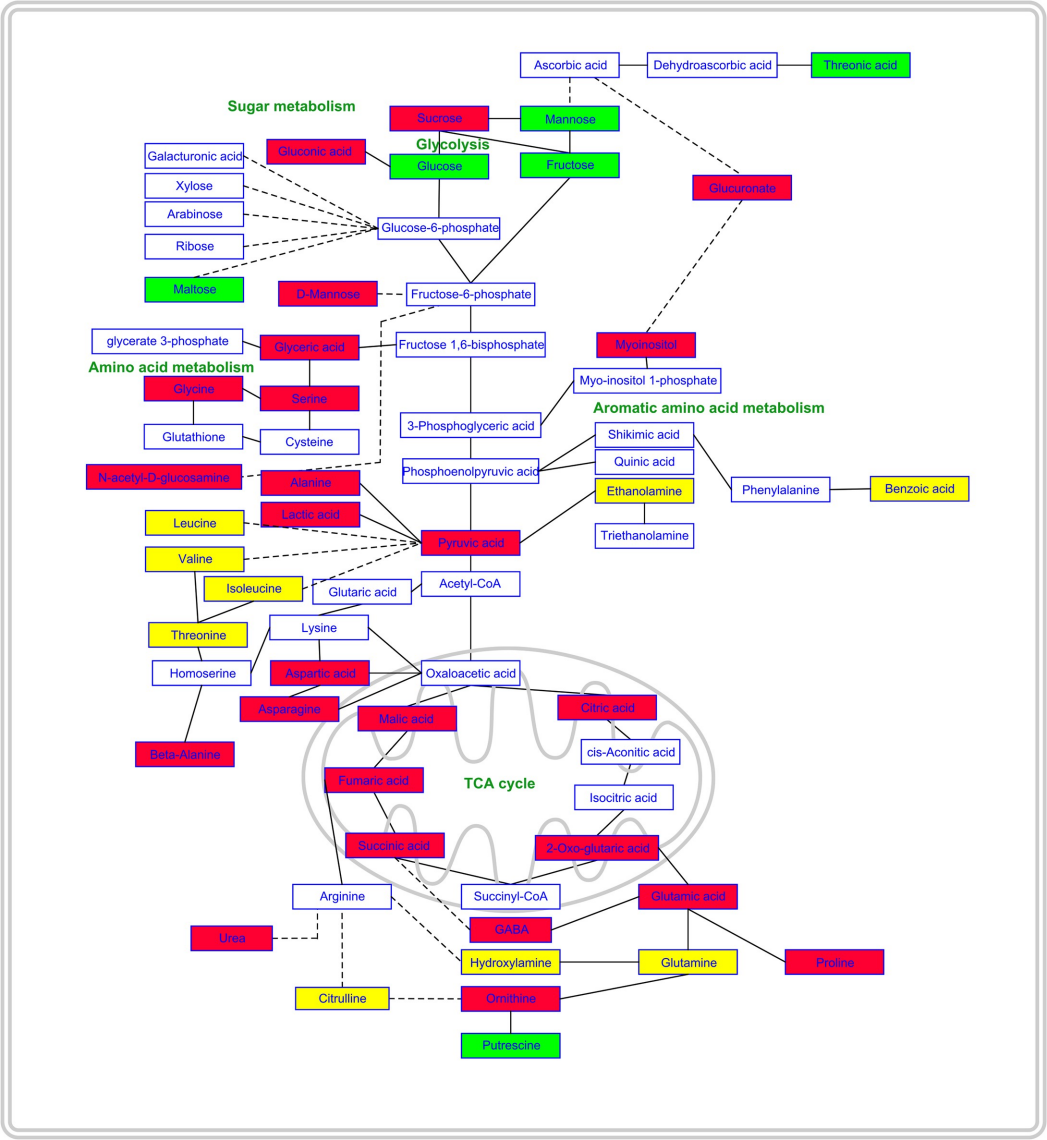
Effects of rosmarinic acid on primary metabolism. Schematic representation of the qualitative changes of metabolite abundance mapped onto the metabolic network. Solid lines in the network indicate a single step connecting two metabolites, whereas dashed lines represent multiple steps. Green filled boxes indicate that, in roots treated, the abundance of the metabolite increased significantly; red filled boxes for decreased metabolites; yellow filled boxes for metabolites which abundance was not significantly affected and empty boxes were used for unmeasured metabolites. The scheme is based on part of the data reported in Table 2. Statistical differences were evaluated through Student’s test with *P* ≤ 0.05. N = 4.

Successively, the mapping of metabolite changes onto the metabolic network highlighted that RA affected distinct localized regions of the cell metabolic network (Fig 9). Regarding sugars content, in RA-treated roots, a decrease in sucrose and an increase in glucose and fructose, linked to the glycolysis pathway, was observed. Moreover, a decrease in myoinositol and glucuronic acid, involved in ascorbic acid metabolism, and an increase in threonic acid, related to ascorbate catabolism, were also detected (Fig 9). Among the compounds linked to 3-PGA, glyceric acid, serine, glycine and glycolic were significantly reduced by the RA-treatment. Similarly, a significant reduction in alanine and lactic acid (linked to pyruvate) as well as aspartic acid and its derived amino acids asparagine, lysine, and β-alanine (linked to oxaloacetic acid) was reported. Concerning the glutamate branch, linked to 2-oxoglutarate, GABA, proline, ornithine and urea were significantly reduced, whereas a significant increment in putrescine was detected. Finally, there was a pronounced reduction in citrate, 2-oxoglutarate, succinate, fumarate and malate (Fig 9).

## Discussion

Rosmarinic acid significantly affected root morphology of *Arabidopsis* seedlings, inducing strong alterations on root cell anatomy and ultrastructure, especially at concentrations higher than 100 μM. In particular, RA inhibited total and primary root length, number and lateral root formation, causing also a reduction of root hairs length and density. This effect was also evident at very low concentrations for a natural product, with an ED_50_ of total root length as low as 71 μM. Microscopy confirmed also root aberrant formations and the loss of root hairs. These effects, already observed in Arabidopsis seedlings exposed to other natural chemicals, such as farnesene, citral, chalcone and scopoletin [8,10,11,34], were evident after 7 days of RA treatment and became significantly stronger after 14 days, underlining a gradual effect of this compound. Transmission electron microscopy (TEM) of RA-treated roots confirmed the root morphological results, revealing important organelle alterations and evident tissue disorganization. Moreover, confocal microscopy experiments pointed out that RA strongly affected cell respiration altering, as highlighted by metabolomic experiments, the TCA cycle and TCA-derived amino acids production.

Similar alterations in root morphology and anatomy were also observed by Pasternak et al. [35] in Arabidopsis treated with alloxan, a known ROS-inducer [36]. For example, primary root deformations observed in RA-treated roots, as well as root hair phenotype (dichotomic root hairs) were a clear symptom of oxidative stress, similar to those observed in the *scn1* mutant, where ROS, especially O_2_^-^, accumulated in ectopic foci and over a greater area of the cell surface compared to wild type [37].

O_2_^-^ and H_2_O_2_ *in-situ* staining confirmed the increase of these ROS in RA-treated root cells, which was also accompanied by a significant inhibition of the first line antioxidants defense represented by SOD and CAT, two ROS scavenging enzymes [38,39]. In particular, SOD is the only plant enzyme capable of scavenging O_2_^-^ [40], whereas CAT catabolizes H_2_O_2,_ detoxifying this compound without any reducing power and providing plants with an energy-efficient way to remove ROS [38,39]. The role of CAT is pivotal in scavenging hydrogen peroxide and its activity inhibition (reduction), despite all the other ROS scavengers, is sufficient to induce H_2_O_2_ accumulation and cell death [41]. Moreover, the phenotype of seedlings treated with NaN_3_, a largely known SOD and CAT inhibitor that was used as positive control, was extremely similar to the phenotype of seedlings treated with RA.

Therefore, the results suggest that RA-treated roots strongly accumulate ROS (i.e. hydrogen peroxide and superoxide), probably due to the inhibition of at least two of the main constitutive scavenging enzymes, responsible for ROS detoxification.

O_2_^-^ is mainly evolved in the complexes I and III of the mitochondrial electron transport chain in non-photosynthetic plant cells [42], and reduced by dismutation to H_2_O_2_, which can cross the mitochondrial membranes reaching other organelles and cells [43,44]. Previous studies have demonstrated that mitochondrial ROS formation can be enhanced in response to different biotic and abiotic stress [45,46] conditions, and in particular to factors (also natural and synthetic chemicals) that inhibited either the cytochrome or the alternative oxidase, as has been demonstrated after the addition of specific inhibitors of these pathways [42,47].

Among the consequences of increased ROS generation in mitochondria are the protein and lipid damages [43].

Peroxidation of mitochondrial membrane polyunsaturated fatty acids by ROS could result in the generation of lipid oxidation products as malondialdehyde (MDA), which increased more than 100% in RA-treated seedlings. Lipid peroxidation caused membrane depolarization and irreversible loss of mitochondrial functions, such as mitochondrial respiration, oxidative phosphorylation and ion transport [48,49]. Actually, mitochondria of RA-treated roots were characterized by a strong reduction of their mitochondrial membrane potential (ΔΨm), already detectable after 7 days and extremely marked after 14 days of treatment. This extreme depolarization of root membranes and the increased MDA content suggest the presence of strong damages on membrane structure and function as previously demonstrated by Frenkel [50].

Damages to proteins, directly by ROS or by lipid peroxidation products, is also another important consequence of mitochondrial dysfunction in plants, usually irreversible and that results in protein denaturation and degradation [43,44].

The presence of RA-induced mitochondrial alterations was further demonstrated by the pathway analysis, which confirmed that TCA cycle is one of the most affected pathways in RA-treated roots. In particular, a significant reduction of citric, 2-oxoglutaric, succinnic, fumaric and malic acids was observed, suggesting, according to the litterature [51,52], that several enzymes involved in the Krebs cycle could be affected by RA-treatment. In fact, Sweetlove et al. [51] observed oxidative stress in Arabidopsis cells treated for 16 hours with the ROS inducer menadione, which affected the mitochondrial electron transport chain and the degradation of several mitochondrial proteins, generally involving subunits of ATP synthase, complex I, aconitase, succinyl CoA ligase, pyruvate and 2-oxoglutarate dehydrogenase complexes.

As previously demonstrated, a reduction of 2-oxoglutarate production causes significant alterations in TCA cycle intermediates, ammonium assimilation and amino acids synthesis [53], which is in accordance with our results. Similar results were previously observed by Baxter et al. [54] in Arabidopsis treated with menadione, which caused an extensive inhibition of the metabolic pathways, including the TCA cycle and several pathways of amino acid metabolism. In fact, RA treatment significantly altered the biosynthetic pathways of different sugars and amino acids such as GABA, glutamic and aspartic acids, serine and glycine.

Significant reduction in sucrose and significant increase in glucose and fructose as well as in threonate, which is also known to be a breakdown product of ascorbate under oxidative stress [55], were also observed in the metabolomic analysis of RA-treated roots. These symptoms are also linked to plant response to oxidative stress. In fact, Xiang et al. [56] demonstrated that during oxidative stress, sucrose can be irreversible cleavaged by invertases to glucose and fructose to be used as carbon and energy sources. The role of the invertases is producing monosaccharides, which play a pivotal role in the protection of plants prior to / or under oxidative stress [57–59].

Hence, the metabolomic pathway analysis allowed us to identify the aminoacidic metabolism and the TCA cycle as the main pathways affected by the RA-treatment. These results, together with the increased depolarization of mitochondrial membrane and the increased MDA content, suggest a consequent impaired energy metabolism due to mitochondrial dysfunction and oxidative damages.

As expected, RA-treated Arabidopsis cells analyzed by transmission electron microscopy (TEM), showed an increase of mitochondrial number as well as higher presence of mitochondria with ultrastructural damages, many of them broken, without cristae, condensed and/or with translucent stroma. These alterations were consistent with damages on both mitochondrial membrane and function. Moreover, similar effects have been observed in roots under other stressing factors [10,34,60]. In particular, the increasing number of dividing mitochondria after RA-treatment suggests a strategy of roots cells to compensate mitochondrial dysfunction and deficit of ATP synthesis [61], as already observed in Arabidopsis root cells treated with other natural compounds such as harpin, coumarins or chalcone [10,62,63].

In addition, changes in the mitochondria induced by increased ROS may produce secondary signals from local (in or close to mitochondria) ROS detection mechanisms that can be transmitted to nuclei [43]. TEM images of RA-treated roots showed a high number of nuclei with irregular shape and highly fragmented and condensed chromatin, as well as an increased number of autophagosomes and autolytic activity suggesting early programmed cell death events [10,64,65]. The extension and distribution of cell death along root meristems, after RA treatment, were detected and confirmed through trypan blue and DAPI staining, and the acridine orange/ethidium bromide double staining allowed us to conclude that cell death in treated roots is mainly due to programmed cell death phenomenon and, in a less extent, to necrosis.

Chemical disruption of mitochondrial function has been previously found to induce programmed cell death (PCD) in a similar way to RA. The cytochrome pathway inhibitor antimycin A induced PCD in oat leaf cells, with the loss of mitochondrial membrane potential, burst of mitochondrial H_2_O_2_ from specific sites, and subsequent chromatin condensation. All these effects were prevented by ROS scavengers [66], suggesting that ROS were the PCD signal [43]. The involvement of mitochondria-mediated H_2_O_2,_ in the induction of PCD response in plants, has been proposed as well by several authors [67–69], and also demonstrated in oat treated with the phytotoxin victorin [70]. The disruption of active mitochondria is a typical feature of the early stages of programmed cell death [66,71], including both changes in membrane potential and alterations in the oxidation–reduction potential of mitochondria. Membrane potential changes are assumed to be due to the opening of the mitochondrial permeability transition pore, which allows the passage of ions and small molecules. The resulting equilibration of ions leads in turn to the decoupling of the respiratory chain and the release of cytochrome c into the cytosol [70,71].

## Conclusion

RA pointed out a strong phytotoxic potential with concentrations similar to or lower than other natural molecules. Moreover, we have been able to identify its potential mode of action.

In particular, our results suggest that rosmarinic acid inhibits two of the main ROS scavenging enzymes, causing strong ROS accumulation that induces alterations on mitochondrial ultrastructure and activity through the dissipation of ΔΨ_m_, TCA-cycle alteration, cell starvation and consequently cell death of *Arabidopsis* seedlings. All these effects result in a strong inhibition of root growth and development after RA treatment, which convert rosmarinic acid in a promising molecule to be further explored for weed management, as other secondary metabolites already used in marketed bioherbicides (i.e. pelargonic acid or eugenol). It should be highlighted that natural herbicides don’t aim to totally eradicate weeds, but they should decrease their competitivity during the most critical phases of crop development. Therefore, the goal of natural compounds with herbicidal activity such as RA is to reduce the weed pressure giving to the crops an important advantage in the competition for resources in the agroecosystem. Further experiments will be necessary to evaluate the RA phytotoxicity on different crops and weeds to determine the most suitable conditions for its bioherbicidal use.

## Supporting information

S1 Fig Effects of rosmarinic acid on root hair. RA-induced alteration on root hairs (dicothomic root hairs). A) Control root; B) RA-treated (50 μM) roots.
(TIF)

S2 Fig Effects of sodium azide (NaN_3_) on root morphology of *A*. *thaliana*. A) dose response effect of Na-N_3_, at different concentrations (0–800 μM), on root morphology of *Arabidopsis* seedlings (should be noted the reduction in primary root length and lateral root number); B) Magnification of the dose dependent effect of Na-N_3_ on root tips (should be noted the anatomical alteration and the root hair density reduction); C) NaN_3_-induced alterations on root hairs (dicothomic and bulbous root hairs) of both Control and treated (25 μM) roots.
(TIF)

S3 Fig In-vitro effects of RA on SOD and CAT activity. Direct effects of RA (175 μM) on the activity of SOD and CAT enzymes isolated from 14 days old roots of untreated seedlings of *A*. *thaliana*.
(TIF)

S4 Fig Pathway analysis. Result from “*Pathway Analysis*” carried on the concentrations of metabolite identified in Arabidopsis roots treated for 14 days with rosmarinic acid (175 μM). A) Summary of pathway analysis carried out with MetPa; B) Results from ingenuity pathway analysis with MetPa. Total Cmpd: the total number of compounds in the pathway; Hits: is the actually matched number from the uploaded data; P value: is the original p value calculated from the enrichment analysis; Impact: is the pathway impact value calculated from pathway topology analysis.
(TIF)
