# Supplementary figures and images for: Rosmarinic acid induces programmed cell death in Arabidopsis seedlings through reactive oxygen species and mitochondrial dysfunction
Source: PLoS One. 2018 Dec 26;13(12):e0208802. doi: 10.1371/journal.pone.0208802 (PMC6306208; doi:10.1371/journal.pone.0208802)

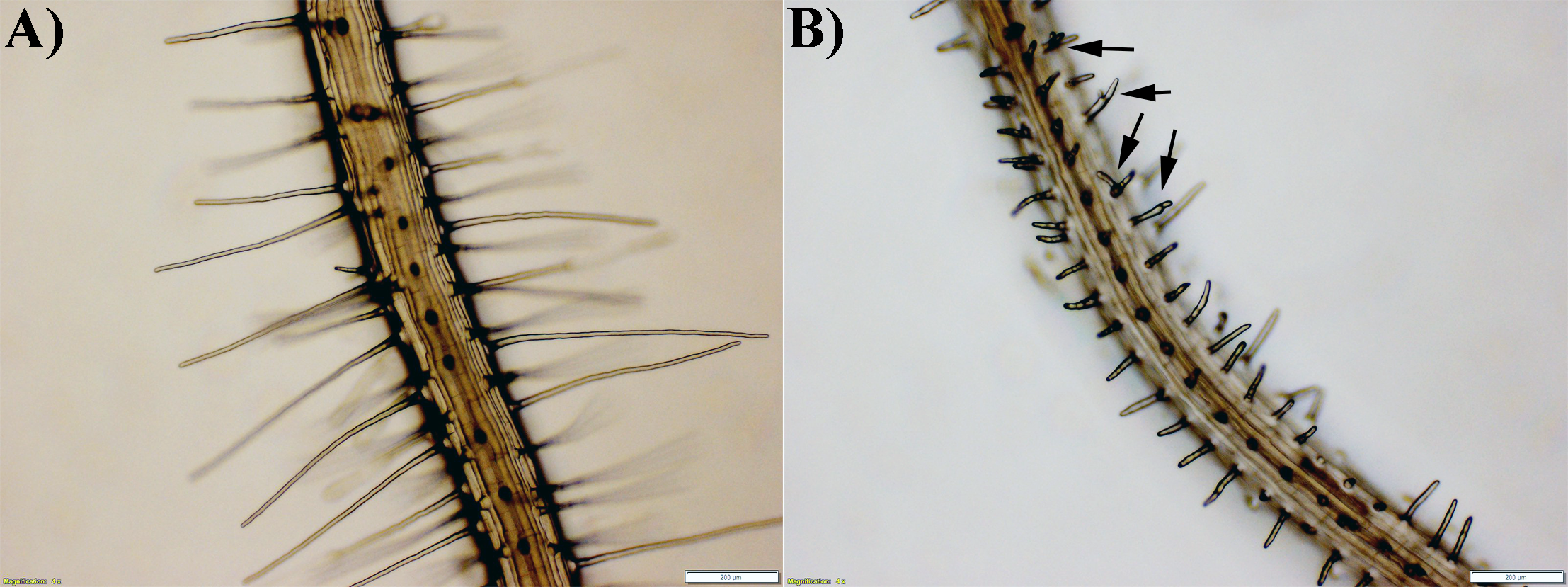

Supplement: S1 Fig — RA-induced alteration on root hairs (dicothomic root hairs). A) Control root; B) RA-treated (50 μM) roots. (TIF) [file pone.0208802.s001.tif]

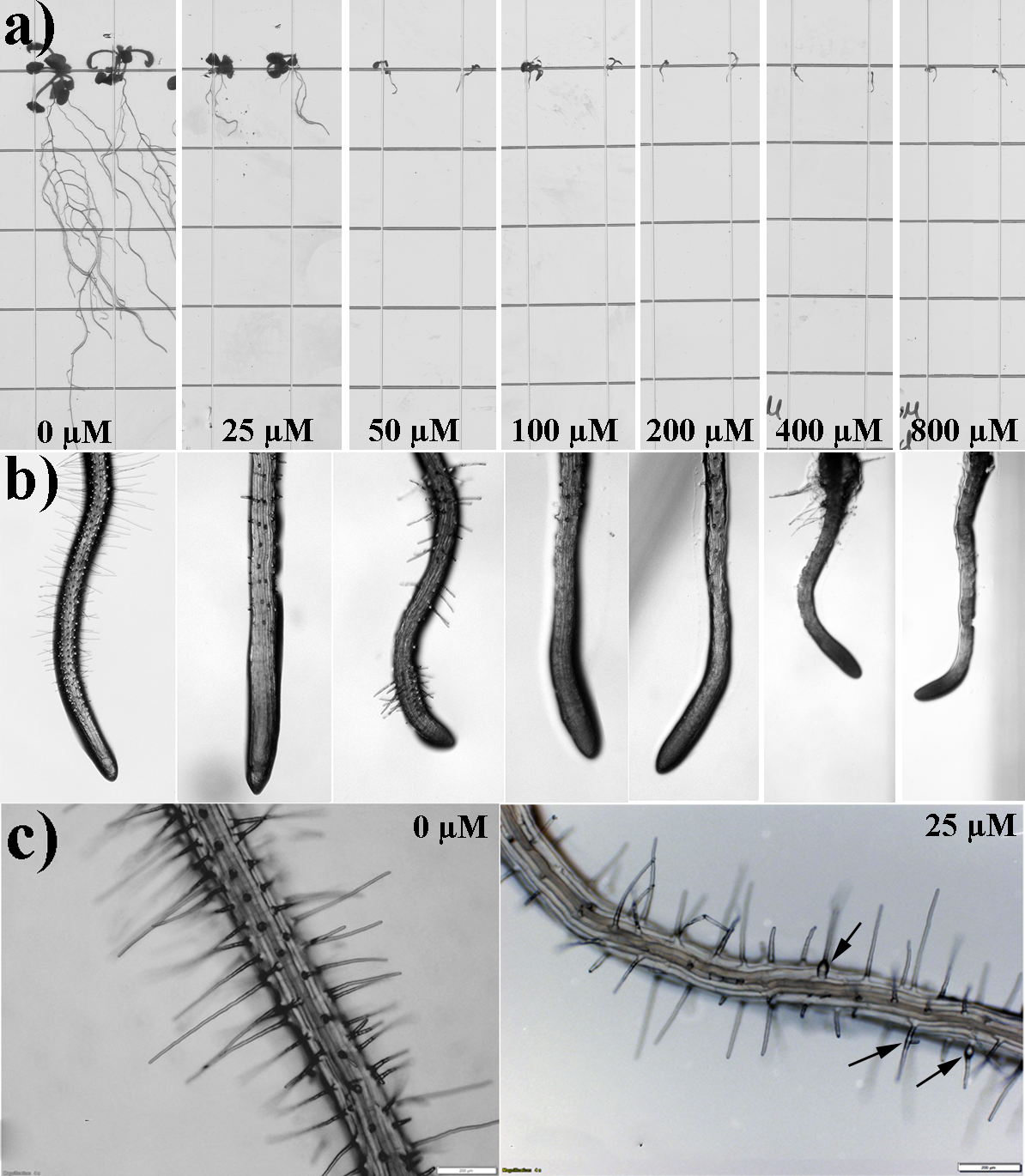

Supplement: S2 Fig — A) dose response effect of Na-N3, at different concentrations (0–800 μM), on root morphology of Arabidopsis seedlings (should be noted the reduction in primary root length and lateral root number); B) Magnification of the dose dependent effect of Na-N3 on root tips (should be noted the anatomical alteration and the root hair density reduction); C) NaN3-induced alterations on root hairs (dicothomic and bulbous root hairs) of both Control and treated (25 μM) roots. (TIF) [file pone.0208802.s002.tif]

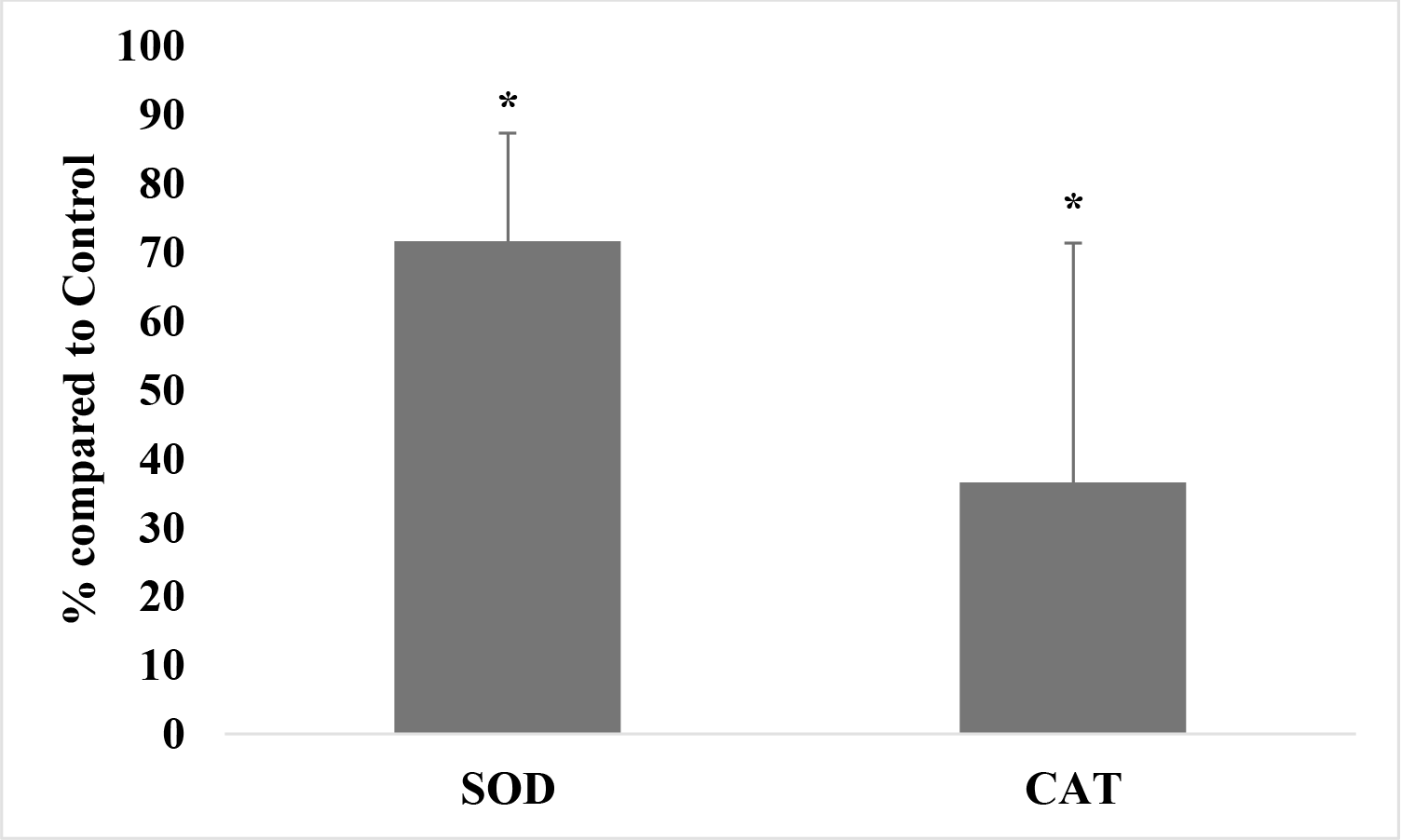

Supplement: S3 Fig — Direct effects of RA (175 μM) on the activity of SOD and CAT enzymes isolated from 14 days old roots of untreated seedlings of A. thaliana. (TIF) [file pone.0208802.s003.tif]

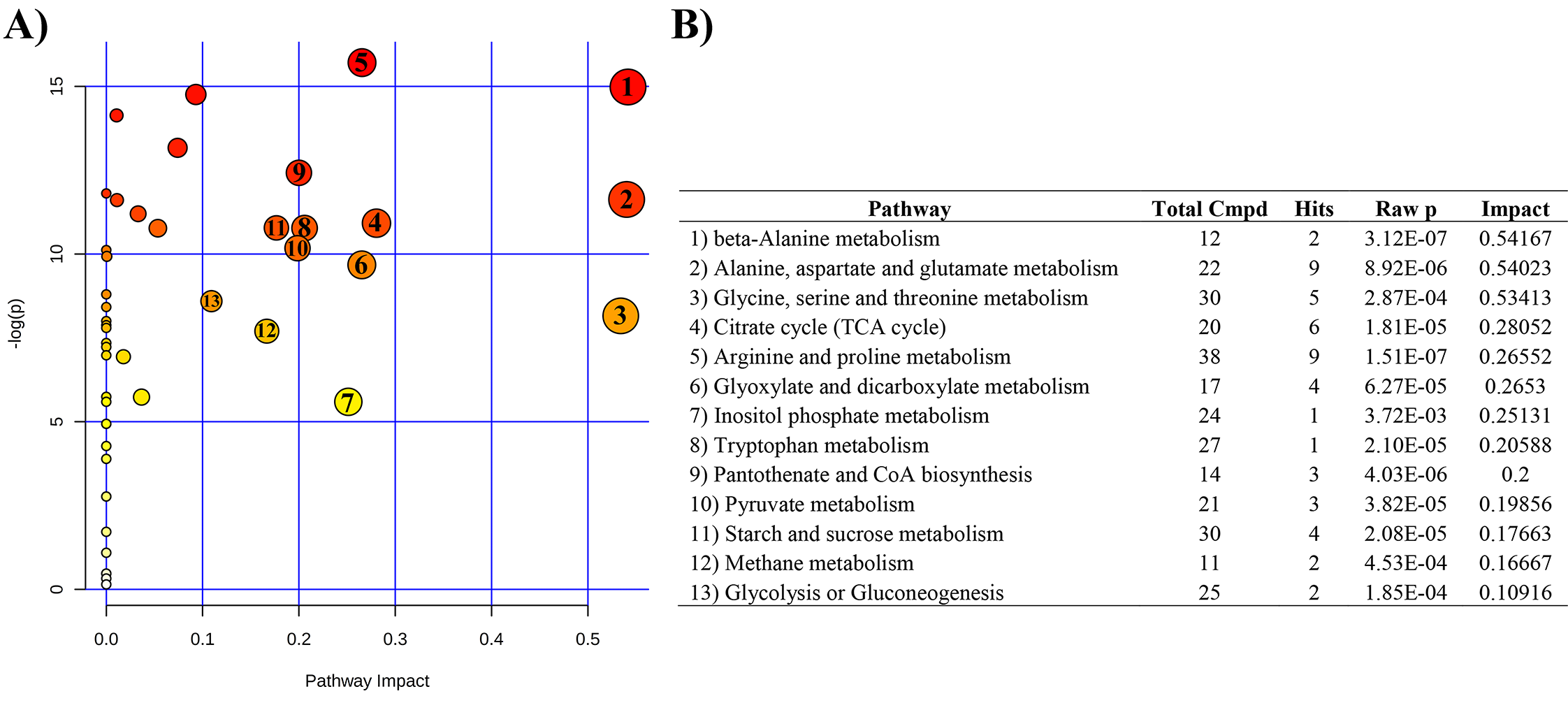

Supplement: S4 Fig — Result from “Pathway Analysis” carried on the concentrations of metabolite identified in Arabidopsis roots treated for 14 days with rosmarinic acid (175 μM). A) Summary of pathway analysis carried out with MetPa; B) Results from ingenuity pathway analysis with MetPa. Total Cmpd: the total number of compounds in the pathway; Hits: is the actually matched number from the uploaded data; P value: is the original p value calculated from the enrichment analysis; Impact: is the pathway impact value calculated from pathway topology analysis. (TIF) [file pone.0208802.s004.tif]
